# Systematic review and comparison of measurement methods for determining the cardiothoracic ratio (CTR) on chest X-rays with subsequent validation using chest CT

**DOI:** 10.1097/MD.0000000000045062

**Published:** 2025-10-03

**Authors:** Dana Belde, Mattias Kettner, Michael J. Thali, Rahel A. Kubik-Huch, André Euler, Tilo Niemann

**Affiliations:** a Department of Radiology, Kantonsspital Baden, Affiliated Hospital for Research and Teaching of the Faculty of Medicine of the University of Zurich, Baden, Switzerland; b Insitute of Forensic Medicine Zurich, University of Zurich, Zurich, Switzerland.

**Keywords:** cardiothoracic ratio, chest x-ray, computed tomography

## Abstract

**Background:**

The cardiothoracic ratio (CTR) is a widely used measurement on chest x-rays (CXR), yet its accuracy and consistency remain limited due to the lack of a standardized measurement method. This study aims to systematically identify and compare established CTR measurement methods from the literature and validate them against computed tomography (CT)-based measurements, which serve as a precise anatomical reference.

**Methods:**

A systematic review was conducted for studies that reported CTR measurement methods on adult CXRs. Eight distinct measurement methods were identified. Five reproducible methods were selected and retrospectively applied to CXRs of 10 patients who also had chest CT scans. The CTR_CXR_ values were compared with CTR_CT_ measurements. Statistical analysis was performed with Friedman test and Dunn–Bonferroni post hoc comparisons.

**Results:**

Systematic literature search yielded in 251 studies of which 38 were included. A total of 8 different measurement methods were identified. Comparative analysis showed significant differences between CTR_CXR_ methods (Friedman test: χ^2^ = 27.104, df = 5, *P* <.001). Method 1 showed a statistically significant underestimation of CTR values compared to CT (adjusted *P* =.004), while Methods 4 and 6 demonstrated the smallest average deviation from CT measurements (3.64% and 4.42%, respectively) and no significant difference (adjusted *P* > .999).

**Conclusion:**

CTR_CXR_ estimation is highly technique-dependent, with considerable variability across published methods. Methods using a midline reference for cardiac measurements and clearly defined anatomical landmarks for assessing thoracic width showed the strongest agreement with CT-based values. The results highlight the need for a standardized and reproducible CTR measurement protocol to ensure consistency and diagnostic accuracy in clinical practice. Based on our analysis, we strongly recommend using a midline reference for cardiac diameter measurements and suggest measuring the thoracic diameter along the internal rib margins at the level of either the left or right diaphragm dome.

## 1. Introduction

The cardiothoracic ratio (CTR) is a traditional chest x-ray (CXR) measurement method used to assess heart size in relation to the chest cavity.^[1,2]^ CXR remains one of the most commonly used imaging techniques,^[3,4]^ making the CTR an accessible and frequently utilized tool for initial cardiac size estimation and the detection of potential cardiomegaly.^[2]^

CTR is commonly defined as the ratio of the maximum cardiac diameter to the maximum inner horizontal diameter of the thoracic cage.^[5,6]^ In posterior–anterior (PA) projection, a standard cutoff value ≥0.5 is commonly employed, with values exceeding this threshold typically interpreted as indicative of cardiac enlargement.^[2,5,7]^

The concept of CTR was first introduced in 1919 by Danzer, who postulated a consistent relationship between heart size and the surrounding thoracic structures.^[8]^ From this perspective, he decided to measure the thoracic cavity at its widest internal rib diameter, typically located “at the level of the apex or one space lower.” Cardiac measurements were accordingly taken at the maximal cardiac width. Using a midline as separator, he measured the left and right borders of the heart separately and then summed both measurements. This result was subsequently divided by the thoracic diameter. Danzer recommended obtaining CXRs at mid-inspiration and considered CTRs exceeding 50% as suspicious for cardiac enlargement.^[8]^

Since then, the CTR has become a widely adopted measurement in CXRs, playing a key role in assessing cardiac size and detecting potential enlargement, despite ongoing controversies concerning its clinical yield.^[9,10]^

Although seemingly straightforward, CTR measurement is influenced by several factors – such as respiratory phase and radiographic projection – which contribute to variability in its application.^[7]^ While most techniques share a common core approach, subtle differences in measurement methods lead to inconsistencies across studies. This lack of a universally standardized definition for CTR presents a significant challenge, resulting in interpretative variability and potentially impacting diagnostic accuracy.

To address this issue and improve the reliability of CTR assessment, this study aims to identify common measurement methods through a systematic literature review and correlate these approaches with computed tomography (CT)-based measurements.

## 2. Materials and methods

The local ethics committee waived clarification of responsibility; only anonymized data sets were used. The systematic review was performed according to the preferred reporting items for a systematic review and meta-analysis (PRISMA) guidelines.^[11]^

### 2.1. Eligibility criteria

Studies assessing the CTR on CXRs (CTR_CXR_) in adult humans were included, regardless of the specific objective of each study. Only studies that provided a description of the methodology for measuring both the thoracic and cardiac diameters were eligible.

Both retrospective and prospective studies were included. Case reports, studies involving pediatric patients, animals, or cadavers were excluded.

### 2.2. Literature search

Literature search was concurrently performed by 2 reviewers in February 2025 using the PubMed database. There was no limit on the dates of publication. Literature search was limited to articles using English language. The following search term was applied: (CXR [tiab] OR chest radiograph [tiab]) AND (CTR [all fields] OR CTR [tiab]) AND (chest [tiab] OR thoracic [tiab] or thorax [tiab]).

### 2.3. Selection process

Retrieved articles were stored in Zotero 6.0.37 reference manager software.^[12]^ The same 2 reviewers independently screened the titles and abstracts to ensure they met the previously mentioned eligibility criteria. Any discrepancies in selection were resolved by consensus.

### 2.4. Data collection process

The 2 reviewers concurrently reviewed the full texts of the eligible studies. Extracted data included authorship, publication year, journal, and the specific method used for CTR measurement. Data were systematically documented in an Excel spreadsheet (Microsoft Corp., Redmond). Regarding CTR measurement, the exact method for determining both the cardiac and thoracic diameters was documented for each study.

### 2.5. Assessment of CTR measurement bias

To systematically and qualitatively assess the reproducibility of the CTR measurement methods,^[13,14]^ the risk of information bias (a subtype of measurement/detection bias) was evaluated for each study.^[11]^ This assessment aimed to determine whether the methods for measuring cardiac and thoracic diameters was described with sufficient clarity and anatomical precision. To the best of our knowledge, no specific tool exists to exclusively evaluate this type of bias. Thus, we performed the risk of bias evaluation similar to domains 2 and 3 of the QUADAS-2 tool for diagnostic accuracy studies.^[15]^

Both reviewers independently evaluated the CTR measurement methods reported in the materials and methods section of each study and reached a consensus on their classification as low, high, or unclear risk of bias. The bias assessment was based on whether the measurement method, specifically the exact definition of cardiac and thoracic measurements, was clearly specified. Studies that provided a detailed description of cardiac and thoracic measurement methods, including specific anatomical reference points, were classified as having low bias. Studies that described cardiac and thoracic measurements and specified anatomical structures but applied a less precise or less detailed method were rated as having unclear bias. Studies that lacked a clear description of the method for measuring cardiac and/or thoracic diameters or did not specify anatomical reference points were classified as having high bias.

### 2.6. Reproduction of CTR measurement methods

According to the bias assessment, CTR measurement methods classified as reproducible (low and unclear bias) were independently applied by both reviewers in a sample of 10 selected patients. Standard CXRs in PA projection between 01.01.2014 and 31.12.2024 were selected. All images had been acquired as part of routine clinical practice, following institutional protocols to ensure consistent patient positioning, source-to-image distance, and exposure parameters. Image review and CTR measurements were conducted using the Picture Archiving And Communication System of our radiology department.

### 2.7. Validation of CTR measurement methods using chest CT

Additionally, we included 10 chest CT scans from the same patients whose CXRs had already been analyzed, ensuring a direct comparison of CTR measurements across both modalities. These CT scans were obtained within a 24-hour interval of the corresponding x-rays.

Chest CT scans were performed using SOMATOM Drive and SOMATOM X.ceed (Siemens Healthineers AG, Erlangen, Germany) following standard institutional protocols as part of routine clinical practice. Images were acquired during end-inspiration with slice thickness set to 1 mm and reconstructed using a soft tissue kernel.

CTR measurements on chest CT were performed within the picture archiving and communication system. The maximum horizontal cardiac diameter was measured on transverse CT images relative to patient frontal plane orientation to ensure accurate assessment of heart size. Similarly, the maximum horizontal inner thoracic cage diameter was measured on transverse CT images to determine the overall thoracic dimensions.

### 2.8. Statistical analysis

We used IBM SPSS Statistics (Version 30.0.0.0, Chicago) and Microsoft Excel 2021 (Microsoft Corp. ) to perform descriptive statistical analyses. Figures included in this manuscript were created using Microsoft PowerPoint 2021 and Microsoft Word 2021 (Microsoft Corp.).

To evaluate differences between the CTR measurement methods, a Friedman test was conducted. Following the significant Friedman result, pairwise comparisons were performed using a Dunn–Bonferroni post hoc test, which adjusts significant values for multiple comparisons to control the family-wise error rate and identify specific methods that differed significantly from one another. The significance level was *P* < .05.

## 3. Results

**Figure 1.**
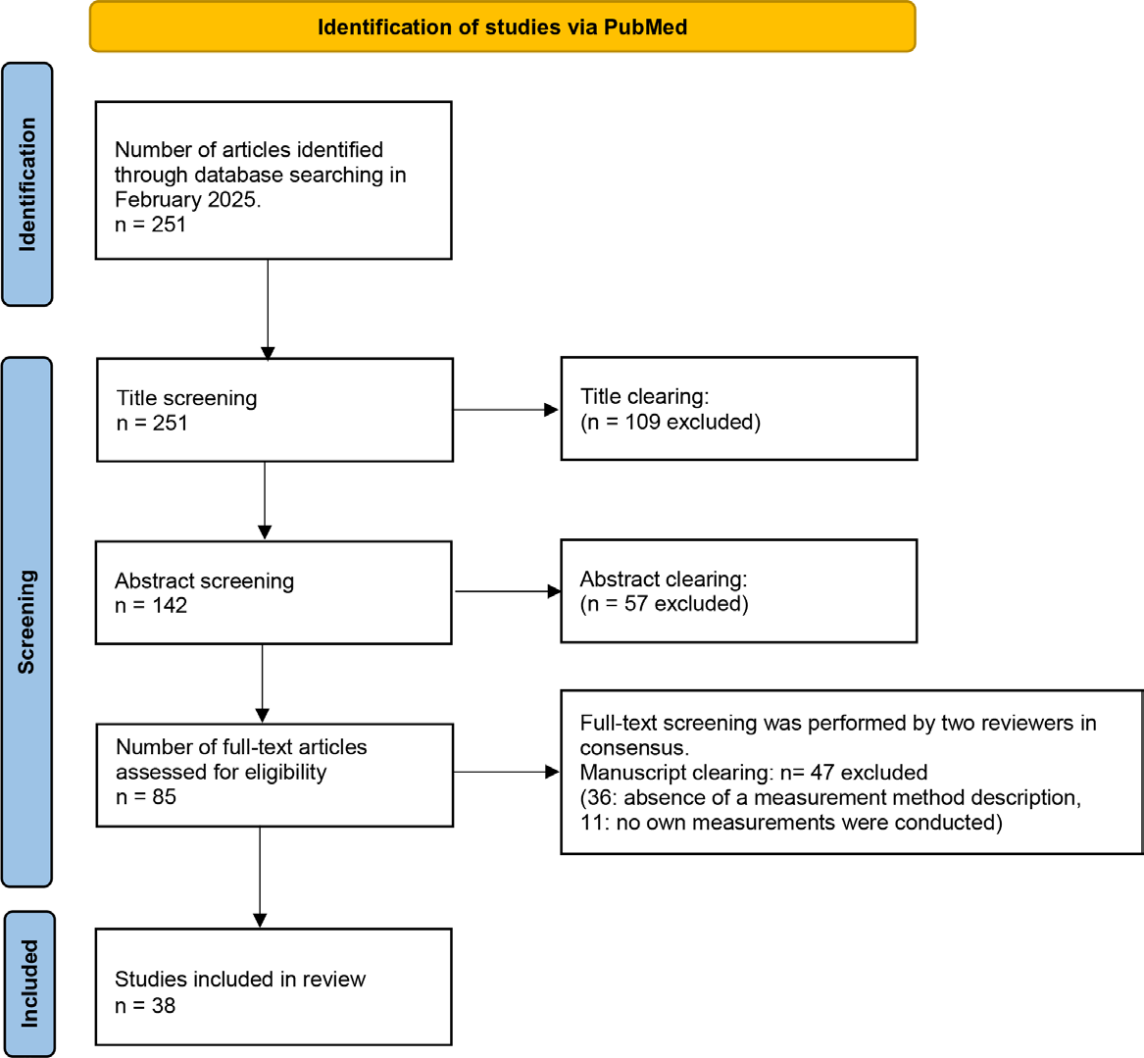
Study selection flow diagram showing a total of 38 included studies.

Literature search resulted in 251 articles. After title and abstract screening, 85 articles were considered as potentially eligible and underwent full-text evaluation. At this stage, 47 articles were excluded, and 38 were ultimately included in the review^[5,6,9,10,16–49]^ (Fig. 1). Detailed description is given in Table S1 (Supplemental Digital Content, https://links.lww.com/MD/Q242).

### 3.1. CTR measurement methods

**Figure 2.**
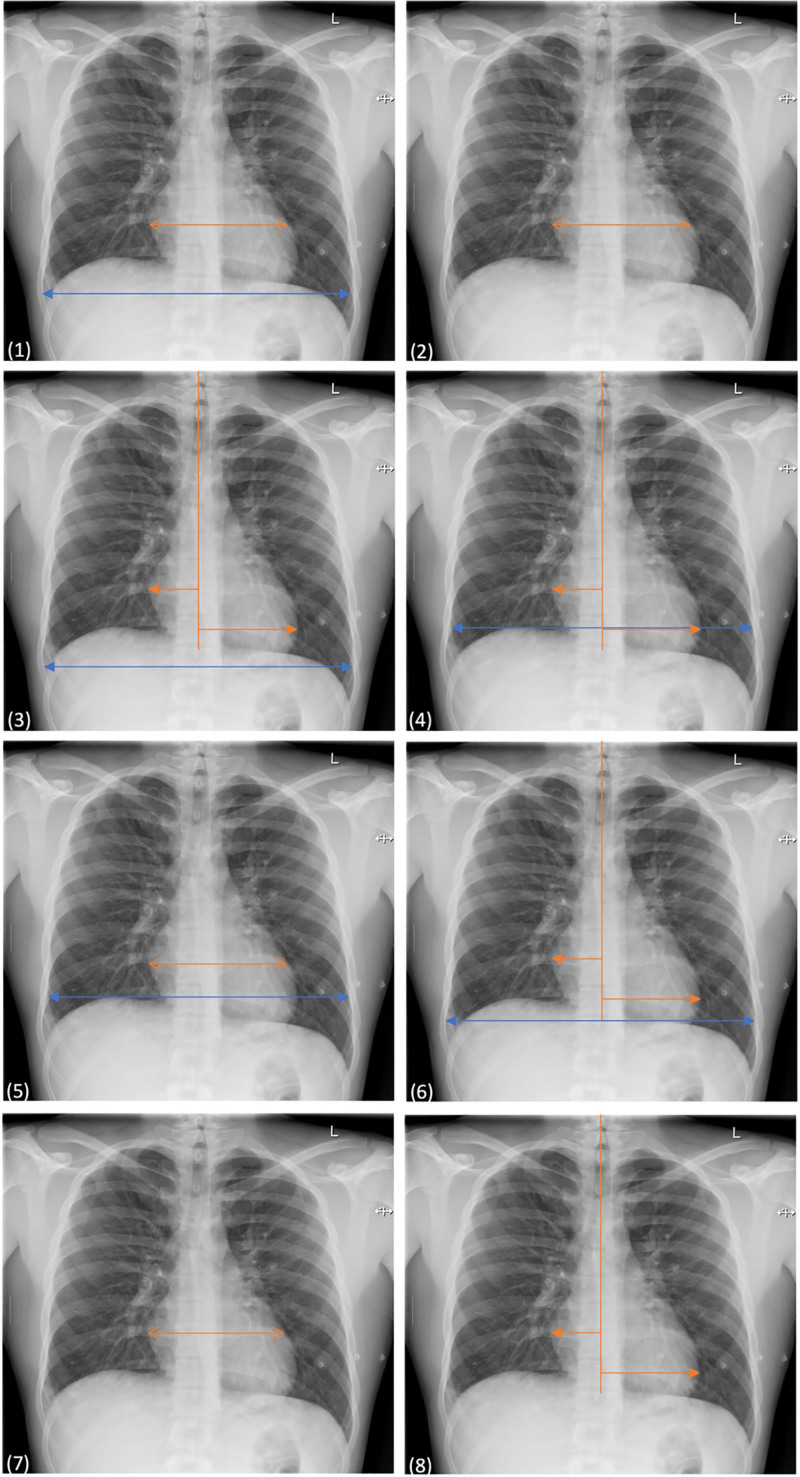
Visualization of the 8 analyzed CTR measurement methods. Methods 2, 7 and 8 are illustrated with cardiac measurements only, as the thoracic measurements were omitted due to their classification as having high risk of bias. Therefore, they were classified as not reproducible. CTR = cardiothoracic ratio.

During full-text evaluation, 8 distinct CTR measurement methods were identified. While all studies determined the CTR as the ratio of the maximum cardiac diameter to the maximum thoracic diameter, differences were noted in how these measurements were defined and performed. The cardiac diameter was measured using different approaches; in some cases, a single continuous horizontal line was used at the widest cardiac diameter, while in others, the left and right heart diameters were measured separately using a midline reference. Furthermore, some authors provided a more detailed definition of thoracic diameter measurement, specifying that it should be taken at the widest internal thoracic diameter, while other studies simply stated that the widest diameter should be measured without further clarification. Additionally, some studies also specified the anatomical level at which the thoracic diameter should be measured (Table 1 and Fig. 2).

**Table 1:** Eight distinct methods for measuring the cardiothoracic ratio were identified.

	Method 1 n = 7	Method 2 n = 11	Method 3 n = 12	Method 4 n = 3	Method 5 n = 2	Method 6 n = 1	Method 7 n = 1	Method 8 n = 1
Cardiac diameter	Maximum transverse cardiac diameter using a single continuous line	Maximum transverse cardiac diameter using a single continuous line	Maximum transverse cardiac diameter measured using a midline as reference, with separate assessments of the left and right heart borders	Maximum transverse cardiac diameter measured using a midline as reference, with separate assessments of the left and right heart borders	Maximum transverse cardiac diameter using a single continuous line	Maximum transverse cardiac diameter measured using a midline as reference, with separate assessments of the left and right heart borders	Maximum transverse cardiac diameter using a single continuous line	Maximum transverse cardiac diameter measured using a midline as reference, with separate assessments of the left and right heart borders
Thoracic diameter	Maximum transverse thoracic internal witdh	Maximum transverse thoracic width	Maximum transverse thoracic internal width	Maximum transverse thoracic internal width on the level of the right diaphragm dome	Maximum transverse thoracic internal width on the level of the right diaphragm dome	Maximum transverse thoracic internal width on the level of the left diaphragm dome	Maximum transverse thoracic width above the level of the diaphragm domes	Maximum transverse thoracic width on the level of the diaphragm domes

Of the 8 different approaches, Method 1 was applied in 7 studies, Method 2 in 11 studies, and Method 3 in 12 studies. Method 4 was used in 3 studies, and Method 5 in 2 studies. Additionally, one study each employed Methods 6, 7, and 8, respectively.

### 3.2. Risk of bias

Among the 8 measurement methods, 3 were classified as having low bias due to clear definitions and well-documented anatomical reference points. 2 methods were rated as having unclear bias due to insufficient detail in their descriptions. Additionally, 3 were assessed as having high bias because they either lacked a precise definition of how cardiac and/or thoracic diameters were measured or failed to specify anatomical reference points.

### 3.3. Reproduction of CTR measurement methods

To reproduce the CTR_CXR_ measurement methods, a case-based analysis was performed comparing the 5 reproducible measurement methods with CT-derived cardiac and thoracic dimensions (CTR_CT_). Table 2 provides an overview of the absolute CTR values obtained using each method.

**Table 2:** Overview of the applied CTR measurement methods and the respective resulting CTR values.

Case	Method 1	Method 3	Method 4	Method 5	Method 6	CT
1	0.49	0.50	0.54	0.53	0.52	0.49
2	0.37	0.37	0.37	0.37	0.37	0.40
3	0.44	0.45	0.46	0.45	0.45	0.46
4	0.40	0.43	0.44	0.41	0.44	0.44
5	0.46	0.49	0.50	0.47	0.49	0.55
6	0.59	0.59	0.61	0.60	0.61	0.60
7	0.53	0.54	0.55	0.54	0.55	0.53
8	0.48	0.48	0.49	0.48	0.48	0.50
9	0.44	0.49	0.52	0.46	0.50	0.52
10	0.45	0.46	0.46	0.45	0.45	0.47

CT = computed tomography, CTR = cardiothoracic ratio.

### 3.4. Validation of CTR measurement methods using chest-CT

A total of 10 patients with corresponding chest CT scans and posteroanterior CXRs were included for comparative analysis of CTR measurement methods. Five different measurement methods were applied to each patient’s CXR, and the resulting CTR values were compared to CT-based measurements, which served as the reference standard. The absolute and percentage deviations from CTR_CT_ values were also calculated for each method. Among the evaluated methods, Method 4 showed the smallest average deviation from the CT reference: 3.6%, followed by Method 3 with 4% and Method 6 with 4.4%. The largest deviations were observed in Method 1 at 6.3% and Method 5 at 6.1% (Table 3).

**Table 3:** Absolute and percentage deviations of the various CTR measurement methods derived from chest x-rays across all cases, compared to CTR values obtained from chest CT.

Case	Method 1	Method 3	Method 4	Method 5	Method 6
1	0 (0%)	0.01 (2.0%)	0.05 (10.2%)	0.04 (8.2%)	0.03 (6.1%)
2	0.03 (7.5%)	0.03 (7.5%)	0.03 (7.5%)	0.03 (7.5%)	0.03 (7.5%)
3	0.02 (4.3%)	0.01 (2.2%)	0.00 (0%)	0.01 (2.2%)	0.01 (2.2%)
4	0.04 (9.1%)	0.01 (2.3%)	0.00 (0%)	0.03 (6.8%)	0 (0%)
5	0.09 (16.4%)	0.06 (10.9%)	0.05 (9.1%)	0.08 (14.6%)	0.06 (10.9%)
6	0.01 (1.7%)	0.01 (1.7%)	0.01 (1.7%)	0 (0%)	0.01 (1.7%)
7	0 (0%)	0.01 (1.9%)	0.02 (3.8%)	0.01 (1.9%)	0.02 (3.8%)
8	0.02 (4.0%)	0.02 (4.0%)	0.01 (2.0%)	0.02 (4.0%)	0.02 (4.0%)
9	0.08 (15.4%)	0.03 (5.8%)	0 (0%)	0.06 (11.5%)	0.02 (3.9%)
10	0.02 (4.3%)	0.01 (2.1%)	0.01 (2.1%)	0.02 (4.3%)	0.02 (4.3%)
Mean	0.03 (6.3%)	0.02 (4.0%)	0.02 (3.6%)	0.03 (6.1%)	0.02 (4.4%)

CT = computed tomography, CTR = cardiothoracic ratio.

The Friedman test revealed a statistically significant difference between at least some of the measurement methods (χ^2^ = 27.104, df = 5, *P* <.001), indicating that the measurement method has a significant impact on CTR_CXR_ values.

Following the significant result of the Friedman test, a post hoc Dunn–Bonferroni test was conducted to perform pairwise comparisons between the individual CTR measurement methods and the CT reference. The analysis revealed that Method 1 differed significantly from the CT-based measurements (adjusted *P* = .004), indicating a systematic underestimation of cardiac size relative to thoracic width. In contrast, Methods 4 and 6 showed the closest agreement with CT-derived values, with adjusted *P*-values of >.999 suggesting no statistically significant difference and therefore better alignment with the reference standard (Table 4).

**Table 4:** Dunn–Bonferroni test results. Demonstrates a significant difference between CTR values measured using Method 1 and the CT-based reference. Also shows that Methods 4 and 6 exhibit the lowest deviation from CT-derived CTR values.

Method - Method	Adj. Significance
1 – CT	.004
3 - CT	.837
4 - CT	.999
5 - CT	.472
6 - CT	.999

CT = computed tomography, CTR = cardiothoracic ratio.

## 4. Discussion

This study conducted a systematic literature review on methods for CTR estimation in CXRs. Eight distinct methods could be identified and analyzed for measuring the CTR on PA CXRs. The accuracy of the 5 reproducible methods was subsequently evaluated against CT-based reference measurements. The findings demonstrate that, although all methods share the common principle of calculating the ratio between cardiac and thoracic widths, there are substantial variations in measurement methods that significantly impact the resulting CTR values.

One of the most striking observations was the methodological heterogeneity in how cardiac and thoracic diameters were defined and measured. Methods 1, 2, 5, and 7 employed a single maximal transverse cardiac diameter, while Methods 3, 4, 6, and 8 used the sum of left and right heart measurements with reference to the spinal midline. The thoracic width was even more variably defined. Methods 1, 3–7 specified the thoracic measurement at the internal rib margins, while others provided only vague instructions. Furthermore, Methods 4–8 indicated the anatomical level at which the measurement should be taken, though the clarity and precision of these descriptions varied (Table 1 and Fig. 2).

As shown in Table 4, Methods 4 and 6, which used the midline as a reference for cardiac measurement, demonstrated better correlation with CT-derived CTR values. This may be attributed to the fact that the more precise cardiac measurement method enables a more reliable and anatomically accurate assessment of cardiac dimensions. The findings also suggest that it is important to clearly state the anatomical level at which the widest thoracic diameter is measured, as Method 4 specified measurement at the level of the right diaphragm dome, while Method 8 used the level of the left diaphragm dome – both within the internal thoracic width. In contrast, Method 1 demonstrated the weakest correlation with CT-based measurements. This may be due to the use of a single continuous line to determine cardiac width and the lack of specification regarding the anatomical level at which the widest thoracic diameter was measured.

It is also notable that the most frequently applied measurement method, Method 3, applied in 12 studies, only showed weak correlation with CT-derived values. Method 2, applied in 11 studies, was classified as having a high risk of bias due to insufficient methodological details, which made reproduction of the measurement method impossible. In this regard, it is worth noting that some studies provided only vague descriptions of their measurement methods and implicitly suggested specific approaches – for example, by illustrating measurements taken within the internal rib margins in their figures without providing a corresponding description in the text.^[9,33]^

As demonstrated in Table 2, case 2 consistently yielded the same CTR_CXR_ value across all measurement methods. This may be attributed to the fact that the patient’s CXR showed no substantial difference between the levels of the left and right diaphragmatic domes. In addition, both domes were visually almost aligned with the apparent maximum inner thoracic diameter – the point most observers probably would intuitively select in the absence of a predefined reference level. Furthermore, the cardiac measurements did not differ substantially between the use of a continuous line and a midline reference. This likely contributed to the consistent values observed across all methods.

Also, it is well-known that CTR measurement is influenced by several factors. One key factor is the respiratory phase, as the heart silhouette appears relatively larger during exhalation and smaller during inspiration.^[7]^ Tomita et al demonstrated that chest dimensions also vary between these phases, resulting in a significantly larger CTR during exhalation.^[50]^ Another important factor is the projection. As previously mentioned, CTR measurements are ideally performed on PA projections, as the heart silhouette is known to appear larger in AP projections.^[7]^

Patient rotation is another factor that can introduce bias in CTR estimation. Sagittal rotation on chest radiographs may cause obliquity in the standard PA projection, potentially altering the cardiac silhouette and impairing accurate assessment of cardiac enlargement. Specifically, right anterior oblique rotation can lead to an underestimation of heart size, whereas left anterior oblique rotation may result in an apparent increase in heart size.^[51]^

The use of parallel lines for measuring the CTR is often implied but not explicitly required in common definitions, which can lead to inconsistencies. The use of parallel reference lines ensures that measurements are taken from anatomically corresponding points on the cardiac borders and the thoracic margins. However, it remains unclear whether parallel lines truly provide the most accurate measurement of CTR, as this approach may not capture the true maximal diameter of the heart or thorax – particularly considering that the heart is a sphere-shaped muscle with asymmetric contours. Additionally, the cardiac axis is not taken into account in presented CTR measurement methods, even though it can significantly affect the apparent width of the heart on CXRs.

The current study has several limitations. The reproduced measurement methods were applied to a relatively small patient sample, which may limit the generalizability of the findings. Furthermore, we only corrected patient frontal plane in CT images as a reference for all size estimations. Additional correction for the true heart axis in chest CT was consciously not performed, as the study was designed to reflect clinical routine as closely as possible. We considered a comparison between axis-corrected CT measurements and CTR values derived from CXRs to be inappropriate, as it would not reflect realistic clinical conditions and could introduce methodological bias. Another limitation of this study is the variation in respiratory phases across the included cases. While the respiratory phase is a known influencing factor, we decided not to include only CXRs and CT scans acquired in full inspiration, since this would also not reflect clinical routine. Instead, we included cases with varying depths of inspiration to better represent the spectrum typically encountered in clinical practice.

## 5. Conclusion

This study identified 8 distinct methods for measuring the CTR on CXRs. Among these, methods that incorporated a midline reference for cardiac measurements and clearly defined anatomical landmarks for thoracic diameter demonstrated the closest alignment with CT-derived reference values. In contrast, commonly used methods that lacked precise measurement definitions showed weaker correlations. These findings highlight the need for a standardized CTR measurement protocol that provides clear and reproducible methodological guidance to improve consistency and diagnostic reliability in clinical practice.

Overall, based on our analysis, we strongly recommend using a midline reference for cardiac diameter measurements and suggest measuring the thoracic diameter along the internal rib margins at the level of either the left or right diaphragm dome.

## Author contributions

**Conceptualization:** Dana Belde, Rahel A Kubik-Huch, André Euler, Tilo Niemann.

**Data curation:** Dana Belde, Tilo Niemann.

**Formal analysis:** Dana Belde, Tilo Niemann.

**Funding acquisition:** Rahel A Kubik-Huch.

**Investigation:** Dana Belde, Tilo Niemann.

**Methodology:** Dana Belde, Rahel A Kubik-Huch, André Euler, Tilo Niemann.

**Project administration:** Rahel A Kubik-Huch, Tilo Niemann.

**Resources:** Rahel A Kubik-Huch.

**Supervision:** Tilo Niemann.

**Validation:** Tilo Niemann.

**Visualization:** Dana Belde, Tilo Niemann.

**Writing – original draft:** Dana Belde, Tilo Niemann.

**Writing – review & editing:** Dana Belde, Mattias Kettner, Michael J Thali, Rahel A Kubik-Huch, André Euler, Tilo Niemann.

## Supplementary Material
