# Supplementary material for: Systematic review and comparison of measurement methods for determining the cardiothoracic ratio (CTR) on chest X-rays with subsequent validation using chest CT
Source: Medicine (Baltimore). 2025 Oct 3;104(40):e45062. doi: 10.1097/MD.0000000000045062 (PMC12499798; doi:10.1097/MD.0000000000045062)
Supplement: Supplementary file 1 [file medi-104-e45062-s001.docx]

**Supplementary Material**

|  |  | **CTR Measuring Method** | |  |  |  |
| --- | --- | --- | --- | --- | --- | --- |
| **Method** | **Author** | **Cardiac evaluation** | **Thoracic evaluation** | **Year** | **Journal** | **Risk of Bias** |
| 1 | Angmorterh, S. K. | maximum transverse cardiac diameter using a single continuous line | maximum transverse thoracic internal witdh | 2024 | BMC Med. Imaging | unclear |
| 1 | Chang, S. A. |  |  | 2022 | Int. J. Cardiovasc. Imaging | unclear |
| 1 | Chou, H.-H. |  |  | 2023 | Diagnostics (Basel) | unclear |
| 1 | Esmail, H. |  |  | 2016 | PLoS ONE | unclear |
| 1 | Hirata, Y. |  |  | 2021 | Can. J. Cardiol. | unclear |
| 1 | Rayner, B. L. |  |  | 2004 | Am. J. Hypertens. | unclear |
| 1 | Simkus, P. |  |  | 2021 | Insights Imaging | unclear |
| 2 | Anjuna, R. | maximum transverse cardiac diameter using a single continuous maximum transverse thoracic witdh | maximum transverse thoracic witdh | 2024 | Curr. Probl. Diagn. Radiol. | high |
| 2 | Bianco, J. A. |  |  | 1980 | Chest | high |
| 2 | Bohn, E. |  |  | 2013 | BMC Nephrol. | high |
| 2 | Dash, H. |  |  | 1980 | Br. Heart J. | high |
| 2 | Lai, S. |  |  | 2015 | Cardiorenal Med. | high |
| 2 | Pudil, R. |  |  | 2007 | Acta Med. (Hradec Králové) | high |
| 2 | Sahin, H. |  |  | 2019 | Int. J. Cardiovasc. Imaging | high |
| 2 | Truszkiewicz, K. |  |  | 2021 | J. Clin. Med. | high |
| 2 | Truszkiewicz, K. |  |  | 2022 | Radiol. Res. Pract. | high |
| 2 | Widhalm, G. |  |  | 2024 | Artif. Organs | high |
| 2 | Zema, M. J. |  |  | 1987 | Br. Heart J. | high |
| 3 | Benge, W. | maximum transverse cardiac diameter using a single continuous line   maximum transverse cardiac diameter measured using a midline as reference, with separate assessments of the left and right heart borders | maximum transverse thoracic internal witdh | 1980 | Circulation | low |
| 3 | Chen, Y.-J. |  |  | 2024 | Biomedicines | low |
| 3 | Debnath, J. |  |  | 2018 | Med. J. Armed Forces India | low |
| 3 | Frishman, W. H. |  |  | 1992 | Am. Heart J. | low |
| 3 | Gao, N. |  |  | 2011 | Clin. Nephrol. | low |
| 3 | Kabala, J. E. |  |  | 1987 | Br. J. Radiol. | low |
| 3 | Khan, A. A. |  |  | 2023 | J. Pak. Med. Assoc. | low |
| 3 | Matusik, P. S. |  |  | 2022 | J. Clin. Med. | low |
| 3 | Okute, Y. |  |  | 2017 | J. Atheroscler. Thromb. | low |
| 3 | Pan, D. |  |  | 2021 | Clin. Res. Cardiol. | low |
| 3 | Park, H. E. |  |  | 2018 | Int. J. Hypertens. | low |
| 3 | Que, Q. |  |  | 2018 | Annu. Int. Conf. IEEE Eng. Med. Biol. Soc. | low |
| 4 | Jun, S. J. | maximum transverse cardiac diameter measured using a midline as reference, with separate assessments of the left and right heart borders | maximum transverse thoracic internal witdh on the level of the right diaphragm dome | 2013 | Int. J. Cardiovasc. Imaging | low |
| 4 | Schmidt, H. C. |  |  | 1996 | Eur. Radiol. | low |
| 4 | van Beers, E. J. |  |  | 2014 | Am. J. Hematol. | low |
| 5 | Hemingway, H. | maximum transverse cardiac diameter using a single continuous line maximum transverse thoracic internal witdh on the level of the right diaphragm dome | maximum transverse thoracic internal witdh on the level of the right diaphragm dome | 1998 | Eur. Heart J. | unclear |
| 5 | Jia, H. |  |  | 2024 | Int. J. Cardiovasc. Imaging | unclear |
| 6 | Madsen, E. B. | transverse cardiac diameter measured using a midline as reference, with separate assessments of the left and right heart borders | maximum transverse thoracic internal witdh on the level of the left diaphragm dome | 1984 | Am. Heart J. | low |
| 7 | Torun, E. | maximum transverse cardiac diameter using a single continuous line | maximum transverse thoracic witdh above the level of the diaphragm domes | 2008 | Eurasian J. Med. | high |
| 8 | Kufel, J. | maximum transverse cardiac diameter measured using a midline as reference, with separate assessments of the left and right heart borders | maximum transverse thoracic witdh on the level of the diaphragm domes | 2024 | J. Clin. Med. | high |

**Table 1.** Overview of bias estimation of all publications and applied CTR measurement methods.
